# Dangers of residual confounding: a cautionary tale featuring cognitive ability, socioeconomic background, and education

**DOI:** 10.1186/s40359-021-00653-z

**Published:** 2021-09-18

**Authors:** Kimmo Sorjonen, Daniel Falkstedt, Alma Sörberg Wallin, Bo Melin, Gustav Nilsonne

**Affiliations:** 1 Division of Psychology, Department of Clinical Neuroscience, Karolinska Institutet, 171 77 Stockholm, Sweden; 2 Institute of Environmental Medicine, Karolinska Institutet, Stockholm, Sweden; 3 Department of Global Public Health, Karolinska Institutet, Stockholm, Sweden; 4 Department of Psychology, Stockholm University, Stockholm, Sweden

**Keywords:** Cognitive ability, Discrimination, Education, Residual confounding, Socioeconomic background, Switching predictors and outcomes

## Abstract

**Background:**

Cognitive ability and socioeconomic background (SEB) have been previously identified as determinants of achieved level of education. According to a “discrimination hypothesis”, higher cognitive ability is required from those with lower SEB in order to achieve the same level of education as those with higher SEB. Support for this hypothesis has been claimed from the observation of a positive association between SEB and achieved level of education when adjusting for cognitive ability. We propose a competing hypothesis that the observed association is due to residual confounding.

**Methods:**

To adjudicate between the discrimination and the residual confounding hypotheses, data from the 1997 National Longitudinal Survey of Youth (NLSY97, *N* = 8984) was utilized, including a check of the logic where we switched predictor and outcome variables.

**Results:**

The expected positive association between SEB and achieved level of education when adjusting for cognitive ability (predicted by both hypotheses) was found, but a positive association between cognitive ability and SEB when adjusting for level of education (predicted only by the residual confounding hypothesis) was also observed.

**Conclusions:**

These results highlight the potential use of reversing predictors and outcomes to test the logic of hypothesis testing, and support a residual confounding hypothesis over a discrimination hypothesis in explaining associations between SEB, cognitive ability, and educational outcome.

## Introduction

Studies have found an association between individuals’ socioeconomic background (SEB) and achieved level of education or socioeconomic position even when adjusting for cognitive ability [e.g. 1, 2]. Some researchers have explained this association with negative social expectations and discrimination against people from humbler origins and favoritism of the highborn. The persistence of this association when adjusting for cognitive ability has been interpreted to mean that higher cognitive ability is required from someone with a lower SEB in order to achieve the same level of education or socioeconomic position as someone with a higher SEB, or alternatively that high SEB can compensate for a lack in cognitive ability [1, 3, 4]. However, other studies have found that when adjusting for achieved socioeconomic position, a positive association between SEB and cognitive ability as well as achieved level of education can be observed [5–10]. If using the same logic as above, a contradictory interpretation would emerge: higher cognitive ability is required from individuals with high SEB in order to achieve the same socioeconomic position as someone with lower SEB, i.e. there is societal discrimination against the highborn.

An alternative explanation, which could account for the above-mentioned seemingly contradictory associations, is residual confounding. Confounding in a statistical analysis occurs when a variable (Z) influences both the dependent variable (Y) and the independent variable (X) [11]. It is common to adjust for potential confounding variables by including them as covariates in an analysis, in order to reduce the risk of spurious associations. However, the influence of the confounding variable may not be fully attenuated by such adjustment [12–16]. Residual confounding refers to confounding which remains despite adjustment. The impact of residual confounding is increased by higher true degree of confounding, larger sample size, and higher reliability in the measurements of X and Y, while it is attenuated by a high reliability in the measurement of Z [12–16]. With these factors in place, even if entities/individuals have the same value on observed Z they will tend to differ in their true Z and this may result in an association between observed X and observed Y even if adjusting for observed Z. For example, even if achieved socioeconomic position or level of education has been rated as the same, the actual/true position or level may be higher for those with high SEB compared to those with lower SEB. Similarly, even if observed cognitive ability is the same, true ability may tend to be higher for those with high SEB. This could explain why high SEB is associated with a higher achieved socioeconomic position and level of education, even when adjusting for observed ability.

The expected standardized effect of measured SEB on true cognitive ability when adjusting for measured cognitive ability is given by Eq. (1) (see “Appendix” for derivation). Assuming that cognitive ability is not measured completely without reliability (*r*_TrCA,CA_ ≠ 0) and that the correlation between observed ability and SEB does not equal unity (*r*_CA,SEB_ ≠ 1), we see that the effect of observed SEB on true cognitive ability when adjusting for observed ability is expected to be zero only if the correlation between observed cognitive ability and SEB equals zero (*r*_CA,SEB_ = 0) or if cognitive ability is measured with perfect reliability (*r*_TrCA,CA_ = 1). Consequently, observed SEB is expected to be associated with whatever true cognitive ability is associated with, e.g. achieved level of education, even when adjusting for measured cognitive ability. It should be noted that in the present context, the term “reliability” should be interpreted more broadly than just, for example, homogeneity. If some research participants would not take the measurement of cognitive ability seriously, e.g. due to low motivation, this could actually strengthen the correlations between scores on subtests and, consequently, the homogeneity of the tests. However, such lack of earnestness among some participants would tend to weaken the correlation between true and measured cognitive ability.

1 $$E|{\beta}_{SEB,TrCA.CA}|=\frac{{r}_{CA,SEB}\times (1-{r}_{TrCA,CA}^{2})}{{r}_{TrCA,CA}\times (1-{r}_{CA,SEB}^{2})}$$

According to a “discrimination hypothesis”, a positive association between SEB and achieved level of education is expected to persist when adjusting for cognitive ability [1, although they do not use the term “discrimination hypothesis”]. However, when adjusting for achieved level of education, the discrimination hypothesis predicts a negative association between SEB and cognitive ability, indicating that higher ability was required from those with lower SEB in order to achieve the same level of education as those with higher SEB. We propose the competing “residual confounding hypothesis”, which implies that any two of cognitive ability, SEB, and achieved level of education will be positively associated even when adjusting for the third, due to imperfect measurement. Furthermore, the discrimination hypothesis predicts that a difference score between achieved level of education and cognitive ability (both variables standardized) will be positively associated with SEB. This difference score is a measure of the degree to which participants are, in a manner of speaking, more educated than intelligent. The residual confounding hypothesis does not imply any association between the difference score and SEB.

### Aims

This study aimed to investigate:

- whether the discrimination hypothesis or the residual confounding hypothesis is best supported by empirical data.
- whether, in the present case, reversing the predictors and outcomes yields a viable test of the logic of inference.

To the best of our knowledge, this is the first explicit investigation of the possibility that adjusted associations between SEB, cognitive ability, and achieved level of education may be due to residual confounding rather than discrimination.

## Method

### Respondents

Publicly available data from the 1997 National Longitudinal Survey of Youth (NLSY97), collected from 8984 US youths (4385 women and 4599 men) born between 1980 and 1984, were used for the present analyses. This dataset is suitable for the present investigation as it is large, nationally representative, contains appropriate measures of all three constructs under investigation, and has been widely used in past research.

### Measurements

Most respondents (complete data available for 7008 individuals) took 12 Armed Services Vocational Aptitude Battery (ASVAB) tests in 1997–1998, when they were between 12 and 18 years old: (1) general science; (2) arithmetic reasoning; (3) word knowledge; (4) paragraph comprehension; (5) numerical operations; (6) coding speed; (7) auto information; (8) shop information; (9) mathematical knowledge; (10) mechanical comprehension; (11) electronics information; (12) assembling objects.

We operationalized SEB as parental income, consistent with common practice in the field [17–19]. Total parental income for the years 1997 and 1998, when the respondents were between 12 and 18 years old, was calculated and the natural logarithm of the mean of these two values was used as the indicator of SEB. An income of zero was treated as a missing value and data were available for 7302 respondents.

In 2017, when they were between 32 and 37 years old, respondents were asked about their highest academic degree received, with the values: (0) None, *n* = 515, (1) General educational development, *n* = 862, (2) High school diploma, *n* = 2692, (3) Associate/junior college, *n* = 598, (4) Bachelor’s degree, *n* = 1352, (5) Master’s degree, *n* = 540, (6) Professional degree/PhD, *n* = 149. Degree was treated as a continuous variable and data were available for 6708 respondents.

### Statistical analyses

Factor scores on the first unrotated factor in an analysis of all 12 ASVAB tests was used as an estimate of the respondents’ cognitive ability. The effects of cognitive ability, SEB (operationalized as parental income, see above), and academic degree on each other were calculated with ordinary least squares regression. All three variables were standardized before the analyses. In an additional analysis, the difference between academic degree and cognitive ability was predicted from SEB. The final sample size for the regression analyses was 4654. Data processing and analyses were conducted with R 4.1.0 statistical software [20] employing the psych package [21]. Data and scripts are available at https://osf.io/cwn5u/.

## Results

Table 1 shows descriptive statistics for SEB, cognitive ability, and academic degree, as well as correlations between these three variables and standardized adjusted regression effects. All correlations and regression effects were positive. Academic degree and cognitive ability were more strongly associated with each other, adjusted or not, than with SEB. We also see that the association between SEB and academic degree when adjusting for cognitive ability was weaker (no overlap of confidence intervals) than the association between SEB and cognitive ability when adjusting for academic degree.

**Table 1:** Descriptive statistics for, correlations between, and regression effects of the study variables

Predictor	*M* (*SD*)	*r*		Standardized adjusted effect (95% CI) on		
		CA	Degree	SEB	CA	Degree
1. SEB	47.0 (45.4)^a^	0.395	0.353	–	0.258 (0.232; 0.285)	0.186 (0.159; 0.214)
2. CA	0 (1)	–	0.508	0.278 (0.249; 0.306)	–	0.429 (0.403; 0.455)
3. Degree	2.54 (1.46)		–	0.200 (0.170; 0.229)	0.428 (0.402; 0.454)	–

^a^Mean of parents’ total income in 1997 and 1998, in 1000 USD. Log-transformed before the analyses

**Fig. 1:**
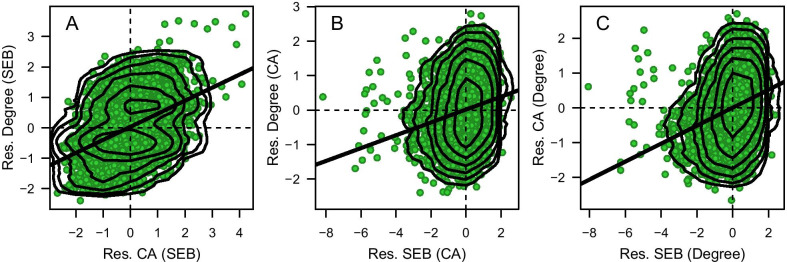
**A** Association between residual cognitive ability and residual academic degree, both adjusted for SEB; **B** association between residual SEB and residual academic degree, both adjusted for cognitive ability; **C** association between residual SEB and residual cognitive ability, both adjusted for academic degree

The adjusted associations, i.e. associations between residuals, are illustrated in Fig. 1. We see that those with higher cognitive ability than predicted from their SEB also tended to have a higher academic degree, and vice versa (panel A); those with higher SEB than predicted from their cognitive ability also tended to have a higher academic degree, and vice versa (panel B); those with higher SEB than predicted from their academic degree also tended to have higher cognitive ability, and vice versa (panel C).

**Fig. 2:**
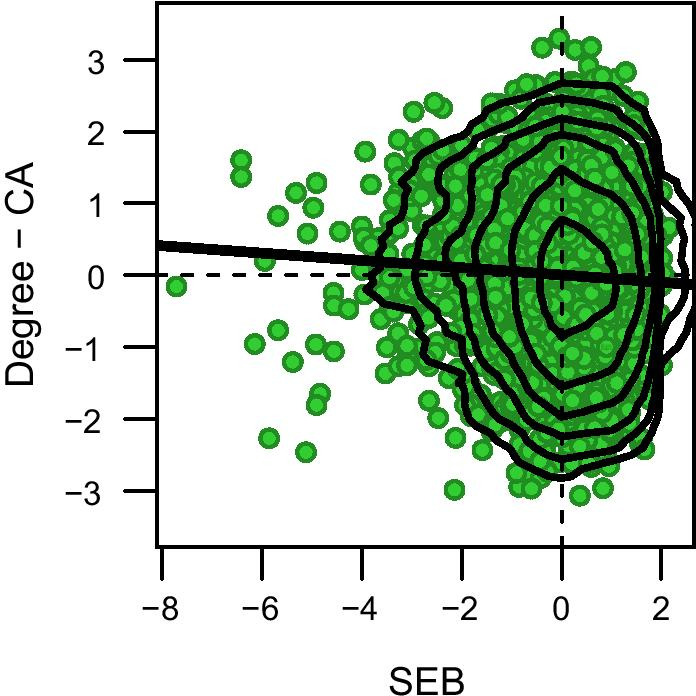
Association between SEB and the difference between academic degree and cognitive ability

The difference between the respondents’ academic degree and cognitive ability was weakly negatively associated with SEB (β =  − 0.051, 95% CI − 0.081; − 0.021, *p* < 0.001, Fig. 2), indicating that those with high SEB did not tend to have a higher standardized score on education than on cognitive ability.

## Discussion

This study aimed to investigate whether the discrimination hypothesis or the residual confounding hypothesis was best supported by empirical data, and whether, in the present case, reversing the predictors and outcomes yielded a viable test of the logic of inference. We show that any two of the variables cognitive ability, SEB, and achieved level of education were positively associated with each other while adjusting for the third variable. A positive association between SEB and education when adjusting for cognitive ability has been observed before and interpreted as indicating that higher cognitive ability is required from those with low SEB in order to achieve the same level of education as those with higher SEB (what we refer to as a discrimination hypothesis, e.g. [1]). However, according to the same logic, the positive association between cognitive ability and SEB when adjusting for level of education would indicate that higher cognitive ability is required from those with high SEB in order to achieve the same level of education as those with lower SEB. In the present data, the adjusted association between cognitive ability and SEB was stronger than the adjusted association between SEB and education. Moreover, and in contradiction to the discrimination hypothesis, no positive association was observed between SEB and the difference between the respondents’ academic degree and their cognitive ability, a measure that indicates to what degree the respondents tend to have a higher standardized score on education than on cognitive ability.

Instead of interpreting these results to indicate simultaneous discrimination of those with low and high SEB, a competing interpretation is that observed associations are due to residual confounding. For example, when adjusting for each other, residual cognitive ability and residual SEB are expected to have, in accordance with Eq. (1), positive associations with their respective true scores and also with the true score on the other variable (e.g. residual SEB has a positive association with true cognitive ability) which, for both variables, results in a positive adjusted association with level of education. Consequently, if two individuals with high and low SEB have the same measured cognitive ability but the former achieves a higher level of education, this does not necessarily indicate that individuals with high and low SEB are privileged and discriminated against, respectively. Alternatively, the former individual may have a higher true cognitive ability, i.e. a more negative residual in measured ability, and this is the reason why he/she achieves a higher level of education.

In the present study, the association between cognitive ability and SEB when adjusting for education was stronger than the association between SEB and education when adjusting for cognitive ability. This does not necessarily indicate that those with high SEB are more discriminated against than those with low SEB. Instead, this discrepancy could be due to lower reliability in the measurement of education compared with the measurement of cognitive ability. Although individuals may formally have achieved the same academic degree there may nonetheless be differences e.g. in the prestige of the university from which they graduated. We predict that on average, those with more prestigious degrees probably have higher SEB and maybe also higher cognitive ability than those with less prestigious degrees.

The present results do not disprove the existence of discrimination on the basis of SEB in processes leading to educational attainment. Rather, the reasoning and evidence presented here is a criticism of one line of evidence that has been advanced in support of a discrimination hypothesis. Causal inference from observational data is fraught with difficulties, of which residual confounding is one [22].

### Limitations

There are several well-established measures of cognitive ability, e.g. the Wechsler Adult Intelligence Scale (WAIS), Raven’s Progressive Matrices (RPM), and the Stanford-Binet Intelligence Scales, and it is possible that these measures would have given slightly different results compared with ASVAB, used in the present study. However, previous research has shown high correlations between different measures of cognitive ability, including Armed Forces Qualification Test (AFQT) score, which is extracted from ASVAB, and classic IQ tests such as California Test of Mental Maturity and Otis-Lennon Mental Ability Test (*r* = 0.81 for both [23]). Parental income represents only one facet of SEB; it is however commonly used and correlated to other facets of SEB such as educational and occupational status [2, 23].

The main message of the present paper is that adjustment for possible confounders can, due to residual confounding, leave room for spurious findings. However, this point does not apply if the observed correlation between the predictor and the possible confounder equals zero or if the possible confounder is measured with perfect reliability (see Eq. (1)). For the application in the present paper, this would mean that with a perfectly reliable measure of cognitive ability, an observed association between SEB and achieved level of education while adjusting for cognitive ability could not be due to residual confounding. The same would be true if the correlation between the measures of cognitive ability and SEB were to equal zero.

## Conclusions

An observed association between two variables, X and Y, while adjusting for a third variable, Z, may be due to residual confounding due to error in the measurement of Z rather than due to a true independent association between X and Y. In our analyses reported here, any two of the variables cognitive ability, socioeconomic background, and achieved level of education were positively associated with each other while adjusting for the third variable. We propose that the most likely explanation for the adjusted associations is residual confounding rather than discrimination. Changing the place of predictors and outcome variables in analyses, to see if results concur with interpretations of the original results, is a simple yet possibly revealing method to validate interpretations. We recommend researchers to use this method and to beware of the dangers of residual confounding.

## Figures and Tables

**Fig. 3:**
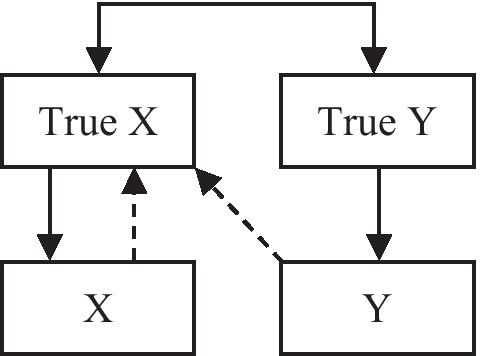
Assumed data generation (solid arrows) and analyzed adjusted effects (dashed arrows)

## Data Availability

The script and data are available at Open Science Framework at https://osf.io/cwn5u/.

## Appendix

The expected effect of Y on True X when adjusting for X is given by (2) [11]:

2 $${\beta }_{Y,TrX.X}=\frac{{r}_{Y,TrX}-{r}_{X,Y}{ \times r}_{TrX,X}}{1-{r}_{X,Y}^{2}}$$

If data is generated as in Fig. 3, correlations between Y and True X and between X and Y, respectively, are expected to be:

3 $${r}_{Y,TrX}={r}_{TrX,TrY}\times {r}_{TrY,Y}$$

4 $${r}_{X,Y}={r}_{TrX,TrY}\times {r}_{TrY,Y}{\times r}_{TrX,X}$$

We can replace terms in (2) with (3) and (4):

5 $${\beta }_{Y,TrX.X}=\frac{{r}_{TrX,TrY}\times {r}_{TrY,Y}-{r}_{TrX,TrY}\times {r}_{TrY,Y}{\times r}_{TrX,X}{ \times r}_{TrX,X}}{1-{r}_{X,Y}^{2}}$$

(5) simplifies to:

6 $${\beta }_{Y,TrX.X}=\frac{{r}_{TrX,TrY}\times {r}_{TrY,Y}\times (1-{r}_{TrX,X}^{2})}{1-{r}_{X,Y}^{2}}$$

We can multiply the numerator and the denominator in (6) by *r*_*TrX,X*_:

7 $${\beta }_{Y,TrX.X}=\frac{{{r}_{TrX,X}\times r}_{TrX,TrY}\times {r}_{TrY,Y}\times (1-{r}_{TrX,X}^{2})}{{r}_{TrX,X}\times (1-{r}_{X,Y}^{2})}$$

The left part of the numerator in (7) equals *r*_*X,Y*_ (see (4)) and we can simplify:

8 $${\beta }_{Y,TrX.X}=\frac{{r}_{X,Y}\times (1-{r}_{TrX,X}^{2})}{{r}_{TrX,X}\times (1-{r}_{X,Y}^{2})}$$

(8) is identical to Eq. (1) in the introduction.
